# Glymphatic Dysfunction in Patients With End-Stage Renal Disease

**DOI:** 10.3389/fneur.2021.809438

**Published:** 2022-01-25

**Authors:** Chang Min Heo, Won Ho Lee, Bong Soo Park, Yoo Jin Lee, Sihyung Park, Yang Wook Kim, Dong Ah Lee, Byeong Cheol Yoo, Kang Min Park

**Affiliations:** ^1^Department of Internal Medicine, Haeundae Paik Hospital, Inje University College of Medicine, Busan, South Korea; ^2^Department of Neurology, Haeundae Paik Hospital, Inje University College of Medicine, Busan, South Korea; ^3^Department of Clinical Research, DEEPNOID, Seoul, South Korea

**Keywords:** chronic kidney disease, diffusion tensor imaging, brain, glymphatic clearance, cerebrospinal fluid (CSF)

## Abstract

**Background:**

We aimed to compare glymphatic dysfunction between patients with end-stage renal disease (ESRD) and healthy controls and analyze the correlation between the glymphatic function and clinical characteristics using the diffusion tensor image analysis along with the perivascular space (DTI-ALPS) index.

**Methods:**

We prospectively enrolled neurologically asymptomatic 49 patients with ESRD undergoing dialysis and 38 healthy controls. Diffusion tensor image was conducted using the same 3T scanner, and the DTI-ALPS index was calculated. We compared the DTI-ALPS index between the patients with ESRD and healthy controls. In addition, we conducted a correlation analysis between the clinical characteristics and DTI-ALPS index in patients with ESRD.

**Results:**

There were significant differences in the DTI-ALPS index between patients with ESRD and healthy controls. The DTI-ALPS index in patients with ESRD was lower than that in healthy controls (1.460 vs. 1.632, *p* = 0.003). In addition, there was a significant positive correlation between the DTI-ALPS index and serum parathyroid hormone levels (*r* = 0.357, *p* = 0.011).

**Conclusion:**

We demonstrated glymphatic dysfunction in patients with ESRD, as revealed by the DTI-ALPS index. This study also reveals the feasibility of the DTI-ALPS method to determine glymphatic function in patients with ESRD, which could be used in future research studies.

## Introduction

The glymphatic system is a highly organized cerebrospinal fluid (CSF) transport system that excretes excess interstitial fluid and metabolic waste products from the brain. Previously, the brain was believed to recycle all protein wastes (1). However, this assumption has been questioned since the discovery of the glymphatic system. Only a small number of proteins are now found to be transported across the blood–brain barrier; this does not include most of the primary proteins that are formed or excreted by the brain cells (2).

Fluid removal from the brain is performed via the glymphatic pathway, a transport system that uses the perivascular space generated by the vascular endfeet of the astrocytes. The astrocyte endfeet, which are in contact with the blood vessel walls of the brain, are composed of the water channel protein aquaporin-4 (AQP-4) (3). Therefore, the glymphatic system is mediated by astrocyte AQP-4 and consists of para-arterial CSF influx, where CSF moves to the brain parenchyma, and paravenous efflux routes, where interstitial fluid moves to the perivenous space. The fluid moved to the perivenous space is finally drained into the cervical lymphatic system. Due to its functional similarity to the peripheral lymphatic system, the astrocyte-regulated mechanism of brain fluid transport is called the “glymphatic (glial-lymphatic) system” (4).

The glymphatic system is known to play an important role in the pathogenesis of certain neurological diseases, such as Alzheimer's disease, traumatic brain injury, and normal pressure hydrocephalus (5, 6). Because arterial pulsation is an important factor in glymphatic influx, arterial stiffening, and cerebral blood flow reduction are closely related to the glymphatic dysfunction (7). The glymphatic system function represents the efficacy of CSF distribution in the brain parenchyma and the clearance rate of parenchymal substrates. Only a few studies have investigated the association between glymphatic dysfunction and neurological disorders owing to limited methods for assessing glymphatic function. MRI with intrathecal gadolinium-based contrast agents is the most representative method to evaluate the glymphatic system (8). However, its clinical utility is limited because of its invasiveness and complexity. Recently, a diffusion tensor image analysis along the perivascular space (DTI-ALPS) index that utilizes non-invasive diffusion images to show glymphatic function has developed. It shows a close relationship with the glymphatic function as assessed with MRI (9). In addition, a study has successfully shown that glymphatic dysfunction in Alzheimer's disease and cognitive function are closely linked with the DTI-ALPS index (10). In addition, a recent study has successfully demonstrated that patients with the cerebrovascular disease have glymphatic dysfunction by the DTI-ALPS method (9).

Patients with end-stage renal disease (ESRD) are known to have a high prevalence of cognitive impairment. Approximately, 16–38% of patients with dialysis have a cognitive impairment, including dementia, which is about three times more prevalent in these patients than in age-matched controls (11). Many patients with ESRD already have a partially damaged cognition when they start dialysis, which further declines during treatment. In particular, impaired executive function in patients with ESRD can suggest early signs of dementia, such as vascular dementia and Alzheimer's disease. In patients undergoing dialysis, a higher risk of stroke, asymptomatic cerebrovascular disease, arteriosclerosis, and increased central pressure have been identified as the major factors affecting cognitive impairment (12). Hemodialysis (HD) itself also causes deterioration of the brain function by inducing uremic toxin retention and repeated cerebral ischemia.

Considering that patients with dementia or cerebrovascular disease have glymphatic system dysfunction, it is judged that patients with ESRD with cognitive impairment or cerebrovascular disease already have glymphatic dysfunction. However, given the fact that ESRD itself has a high probability of developing dementia or stroke in the future, even patients with ESRD without cognitive impairment or cerebrovascular disease can suspect that glymphatic dysfunction already exists. Thus, we can assume that glymphatic dysfunction is present in patients with ESRD, even in the absence of other neurological diseases.

In this study, we aimed to compare glymphatic dysfunction between neurologically asymptomatic patients with ESRD and healthy controls and analyze the correlation between the glymphatic function and clinical characteristics using the DTI-ALPS index.

## Methods

### Participants

This study was conducted with the approval of the institutional review board. The written informed consent was obtained from all the participants. This study was performed prospectively at a single tertiary hospital. We enrolled neurologically asymptomatic 49 patients with ESRD undergoing dialysis from October 2018 to March 2021. The inclusion criteria were as follows: (1) a Glomerular Filtration Rate <15 ml/min/1.73 m^2^, necessitating renal replacement therapy; (2) HD or peritoneal dialysis (PD) for more than 3 months; and (3) no previous history of neurological or psychiatric disorders, which could affect the glymphatic system. We excluded patients with ESRD with structural brain lesions, such as stroke, tumor, or trauma to the brain, and cognitive impairment, which was evaluated with the Korean version of the Consortium to Establish a Registry for Alzheimer's Disease Assessment Packet (CERAD-K) (13) by a board-certified clinical neuropsychologist. Cognition was considered impaired if scores were more than the 1.5 standard deviations below the age- and education-adjusted norms. Patients were considered cognitive impairment if at least two cognitive domains were impaired on the CERAD-K. We obtained the clinical characteristics of the patients with ESRD, such as age, sex, dialysis duration (time interval between the onset of renal replacement therapy and MRI taken), types of renal replacement therapy, and laboratory data.

We also enrolled an age- and sex-matched control group of 38 healthy subjects without any significant past medical, neurological, or psychiatric history. All the healthy controls also had normal brain MRI without structural lesions.

### Diffusion Tensor Imaging (DTI) Acquisition

Diffusion tensor imaging was conducted using the same scanner (3.0 T, 32-channel head coil, AchievaTx, Phillips Healthcare, Best, The Netherlands). It was performed using spin-echo single-shot echo-planar pulse sequences with a total of 32 different diffusion directions (repetition time/echo time = 8,620/85 ms, flip angle = 90°, slice thickness = 2.25 mm, acquisition matrix = 120 × 120, field of view a = 240 × 240 mm^2^, and *b*-value = 1,000 s/mm^2^).

### Diffusion Tensor Imaging Processing

We used the DSI studio program (version 2021 May, http://dsi-studio.labsolver.org) for preprocessing of brain MRI, which included open-source imaging, correction of the eddy current, and phase distortion artifact, setting up a mask (thresholding, smoothing, and defragmentation), and reconstruction with the DTI method.

### DTI-ALPS Index

We drew a rectangular region of interest (ROI) in which the lateral projections of the medullary veins traced orthogonal to the primary diffusion directions. Then, we obtained the fiber orientation and diffusivities of the three directions along the x-, y-, and z-axes as voxel levels at the ROI (Supplementary Figure 1). Of the several voxels, we selected one voxel for each fiber on the same x-axis (projection, association, and subcortical fibers), which showed the maximum orientation in each fiber. The DTI-ALPS index was calculated using the following formula (10, 14, 15):


$$\begin{array}{l} \text{ALPS index }=\;\frac{\text{mean }\left(\text{Dxxproj},\text{ Dxxassoci}\right)}{\text{mean }\left(\text{Dyyproj},\text{ Dzzassoci}\right)} \end{array}$$

Dxxproj: diffusivity along the x-axis in the projection fiber, Dxxassoci: diffusivity along the x-axis in the association fiber, Dyyproj: diffusivity along the y-axis in the projection fiber, Dzzassoci: diffusivity along the z-axis in the association fiber.

### Statistical Analysis

Statistical comparisons were conducted using the chi-square test for categorical variables and the independent samples *t*-test for continuous variables. Correlation analysis was performed using Pearson's correlation coefficient. Statistical significance was defined as a two-tailed *p* < 0.05. Multiple corrections were applied when we conducted statistical analysis for diffusivities along the axis in the fibers (Bonferroni correction, *p* = 0.0055 [0.05/9]). All statistical analyses were performed using MedCalc® Statistical Software version 20 (MedCalc Software Limited, Ostend, Belgium; https://www.medcalc.org; 2021).

## Results

### Clinical Characteristics of the Patients With ESRD

Table 1 shows the clinical characteristics of patients with ESRD. Of the 49 patients with ESRD, 28 underwent HD as renal replacement therapy, whereas 21 patients underwent PD. The patients with ESRD and healthy controls were age and sex matched (60 vs. 61 years, *p* = 0.499; 28/49 [57%] vs. 21/38 [55%], *p* = 0.861, respectively).

**Table 1:** Clinical characteristics of the patients with end-stage renal disease (ESRD).

**Clinical data**	**Patients with ESRD (*N* = 49)**	**Healthy controls (*N* = 38)**	***P*-value**
**Demographic data**			
Age, years (SD)	60.0 (8.1)	61.1 (7.5)	0.499
Men, *N* (%)	28 (57.1)	21 (55.2)	0.861
Hemodialysis, *N* (%)	28 (57.1)		
Dialysis duration, months (SD)	38.6 (48.4)		
**Laboratory data**			
Hemoglobin, g/dL (SD)	10.2 (1.2)		
Hematocrit, % (SD)	31.6 (3.6)		
Protein, g/dL (SD)	6.4 (0.6)		
Albumin, g/dL (SD)	4.3 (4.2)		
Aspartate aminotransferase, U/L (SD)	20.3 (6.9)		
Alanine aminotransferase, U/L (SD)	17.7 (9.5)		
BUN, mg/dL (SD)	56.4 (18.0)		
Creatinine, mg/dL (SD)	8.6 (2.5)		
Sodium, mmol/L (SD)	139.2 (3.4)		
Potassium, mmol/L (SD)	4.6 (0.7)		
Chloride, mmol/L (SD)	100.0 (4.2)		
Calcium, mg/dL (SD)	8.3 (0.8)		
Phosphate, mg/dL (SD)	4.6 (1.0)		
Parathyroid hormone, pg/mL (SD)	313.9 (265.9)		
Total CO_2_ contents, mmol/L (SD)	24.1 (4.2)		

### DTI-ALPS Index

**Figure 1:**
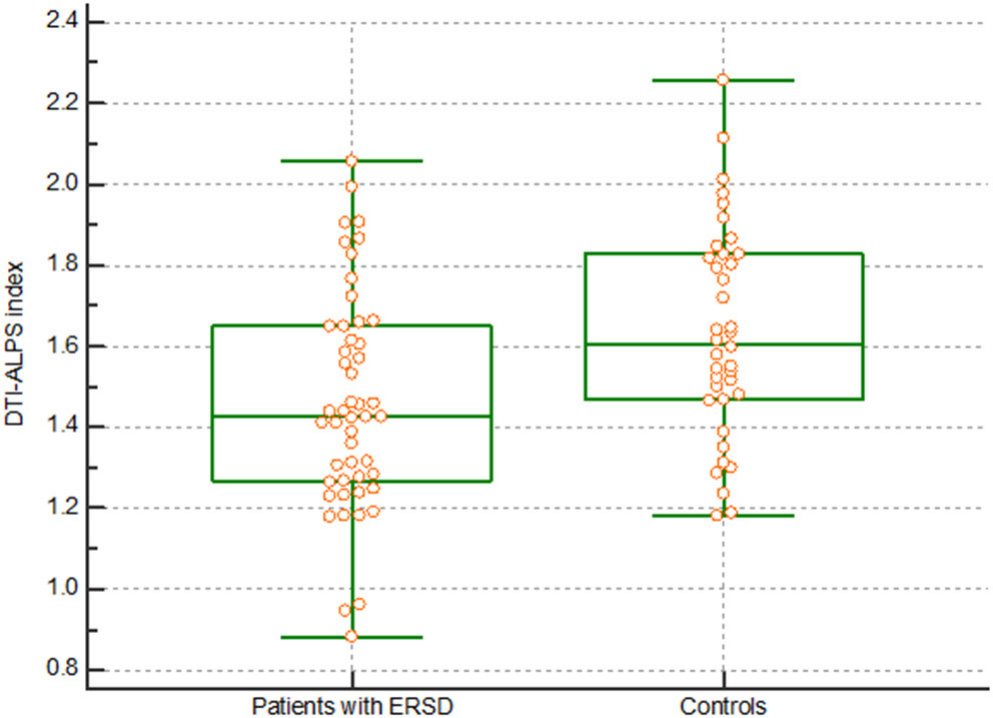
DTI-ALPS index. The graph shows a significantly lower DTI-ALPS index in patients with ESRD than in healthy controls. DTI-ALPS, diffusion tensor image analysis along with the perivascular space; ESRD, end-stage renal disease.

There were significant differences in the DTI-ALPS index between patients with ESRD and healthy controls. The DTI-ALPS index in patients with ESRD was lower than that in healthy controls (1.460 vs. 1.632, *p* = 0.003) (Figure 1). In addition, there were differences in the diffusivity along the x-axis, y-axis, and z-axis of the fibers (Supplementary Figure 2).

### Correlation Analysis

**Figure 2:**
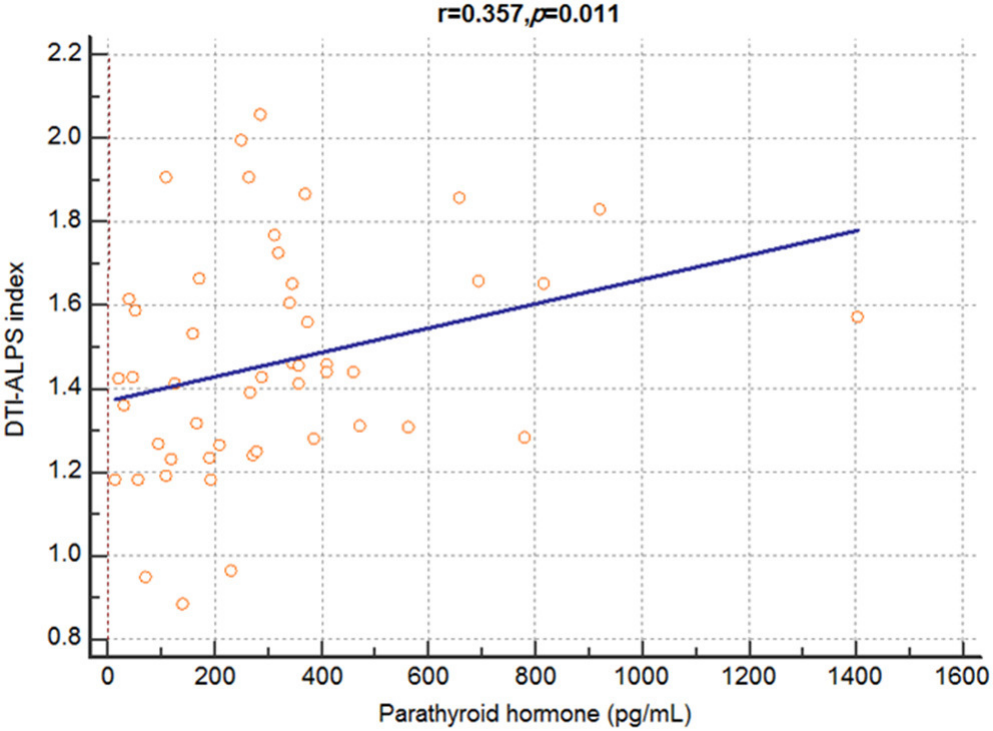
Correlation analysis between DTI-ALPS index and serum parathyroid hormone levels in patients with dialysis. A significant positive correlation is observed between the DTI-ALPS index and serum parathyroid hormone levels in patients with end-stage renal disease. DTI-ALPS, diffusion tensor image analysis along with the perivascular space.

There was a significant positive correlation between the DTI-ALPS index and serum parathyroid hormone (PTH) levels (*r* = 0.357, *p* = 0.011) (Figure 2). However, there was no significant correlation between the DTI-ALPS index and other clinical characteristics, including age (*r* = −0.148, *p* = 0.170); dialysis duration (*r* = 0.059, *p* = 0.686); and serum levels of alanine aminotransferase (*r* = 0.108, *p* = 0.458), albumin (*r* = −0.124, *p* = 0.395), aspartate aminotransferase (*r* = 0.001, *p* = 0.993), urea nitrogen (*r* = 0.067, *p* = 0.646), calcium (*r* = 0.089, *p* = 0.542), chloride (*r* = 0.074, *p* = 0.614), creatinine (*r* = 0.075, *p* = 0.609), phosphate (*r* = 0.142, *p* = 0.329), potassium (*r* = −0.079, *p* = 0.589), protein (*r* = 0.148, *p* = 0.310), and sodium (*r* = 0.109, *p* = 0.454); hematocrit (*r* = 0.131, *p* = 0.370); hemoglobin (*r* = 0.153, *p* = 0.294); and total carbon dioxide (CO_2_) content (*r* = 0.004, *p* = 0.980).

## Discussion

Neurological complications and brain abnormalities are very common in patients with ESRD (16). ESRD is associated with vascular problems and has a higher prevalence of the cardiovascular disease. The brain and kidney are composed of many vascular structures, are exposed to a large amount of blood flow, and are vulnerable to damage as they are end organs. Previous studies have investigated the association between cerebrovascular hemodynamics and brain dysfunction, and significant associations have been demonstrated (17). In addition, a close relationship between vascular disease and increased uremic metabolites has been found in previous studies (18, 19). However, as vascular disease alone cannot explain the brain dysfunction and other neurological abnormalities in patients with ESRD, many studies have been conducted to identify other possible mechanisms underlying such abnormalities. Our previous study using graphic theoretical analysis showed that patients with ESRD had decreased structural and functional brain connectivity, and there was an association between brain connectivity and cognitive function (20). There were even changes in structural connectivity in asymptomatic patients with early chronic kidney disease (CKD) (21). In addition, cognitive decline in patients with ESRD improved after kidney transplantation (22). Risk factors that explain brain dysfunction in patients with ESRD include traditional and non-traditional risk factors. Traditional risk factors, such as aging, diabetes mellitus, and hypertension induce vascular injury, and non-traditional risk factors, such as chronic inflammation, hypercoagulable state, and oxidative stress induce vascular injury, endothelial dysfunction, and neurotoxicity (23). In addition, uremic toxins are associated with vascular injury and endothelial dysfunction, and induce direct neuronal toxicity (24). These risk factors together cause brain damage. The present study revealed a lower DTI-ALPS index in patients with ESRD than in healthy controls. Thus, glymphatic dysfunction may be one of the pathophysiological mechanisms for cognitive impairment and cerebrovascular disease in patients with ESRD.

Uremic toxins and various vascular distresses might have affected the glymphatic system of the brain according to the brain interstitial space and glial cell damage. In this study, we found a positive correlation between metabolic panels, PTH level, and glymphatic function. In CKD, PTH release is controlled by negative feedback regulation by circulating Ca^2+^ levels. We cannot conclude any causal relationship where PTH has a protective role for a better glymphatic system. PTH might be elevated to increase intracellular calcium levels for better signal transduction of AQP-4 because PTH crosses the blood-brain barrier and its receptors are found in the human brain (25). However, PTH levels in patients with dialysis might vary according to their mineral status and drugs that could affect its secretion. In addition, some small case studies revealed that hyperparathyroidism might be associated with cognitive impairment and dementia (26–28). Fibroblast growth factor 23 (FGF23) is also closely related to the PTH level. The level of FGF23, which can alter PTH level by decreasing PTH gene expression (29), is usually high in patients with dialysis due to its receptor resistance. High FGF23 may impact neurologic function by means of promotion of vascular disease or through direct effects (30). Furthermore, a cross-sectional study with 263 prevalent patients with HD showed high FGF23 that may have contributed to cognitive impairment (31). However, elderly patients with CKD not on dialysis showed no relationship between high FGF23 and cognitive functional capacity (32), and an animal study showed that FGF23 deficiency impairs hippocampal-dependent cognitive function (33). Persistent FGF 23 is one cause of secondary hyperparathyroidism in CKD as a long-term inducer of parathyroid cell proliferation and PTH secretion (34). Therefore, we could not determine a conclusive association between the glymphatic function and PTH because of its diversity and unexpected roles in patients with ESRD.

This is the first study to have evaluated glymphatic function in patients with ESRD and to have successfully demonstrated glymphatic dysfunction in these patients. This study will also be meaningful in that it provides a basis for explaining the mechanisms of neurological abnormalities in patients with ESRD. However, this study has some limitations. The sample size was relatively small. We also did not evaluate the difference in dialysis modality owing to the sample size, which might have affected the glymphatic function because patients with HD are more vulnerable to circulation problems than PD patients. Additionally, we could not determine the exact mechanisms for the significant correlation between PTH level and DTI-ALPS index. Lastly, although we excluded the patients with the previous history of neurological disorders or structural brain lesions, we could not evaluate the cerebral artery status, such as atherosclerosis and stenosis, which might be related to glymphatic system function. Despite these limitations, this study has many clinical implications with pioneering results for investigating neurological abnormalities in patients with ESRD.

## Conclusion

We demonstrated glymphatic dysfunction in patients with ESRD using the DTI-ALPS index. This study also reveals the feasibility of the DTI-ALPS method to determine glymphatic function in patients with ESRD, which could be used in future research studies.

## Data Availability Statement

The original contributions presented in the study are included in the article/Supplementary Material, further inquiries can be directed to the corresponding author/s.

## Ethics Statement

The studies involving human participants were reviewed and approved by Institutional Review Board of Haeundae Paik hospital. The patients/participants provided their written informed consent to participate in this study.

## Author Contributions

CH and WL participated in data collection, data interpretation, and manuscript writing. BP participated in data collection, data interpretation, and report writing. YL, SP, YK, DL, and BY participated in data collection and data interpretation. KP supervised analysis, participated in study design, data interpretation, and manuscript writing. All authors provided critical feedback, read, and approved the final manuscript.

## Conflict of Interest

BY was employed by DEEPNOID. The remaining authors declare that the research was conducted in the absence of any commercial or financial relationships that could be construed as a potential conflict of interest.

## Publisher's Note

All claims expressed in this article are solely those of the authors and do not necessarily represent those of their affiliated organizations, or those of the publisher, the editors and the reviewers. Any product that may be evaluated in this article, or claim that may be made by its manufacturer, is not guaranteed or endorsed by the publisher.
