# Supplementary material for: Glymphatic Dysfunction in Patients With End-Stage Renal Disease
Source: Front Neurol. 2022 Jan 25;12:809438. doi: 10.3389/fneur.2021.809438 (PMC8821099; doi:10.3389/fneur.2021.809438)
Supplement: Supplementary file 1 [file Data_Sheet_1.PDF]

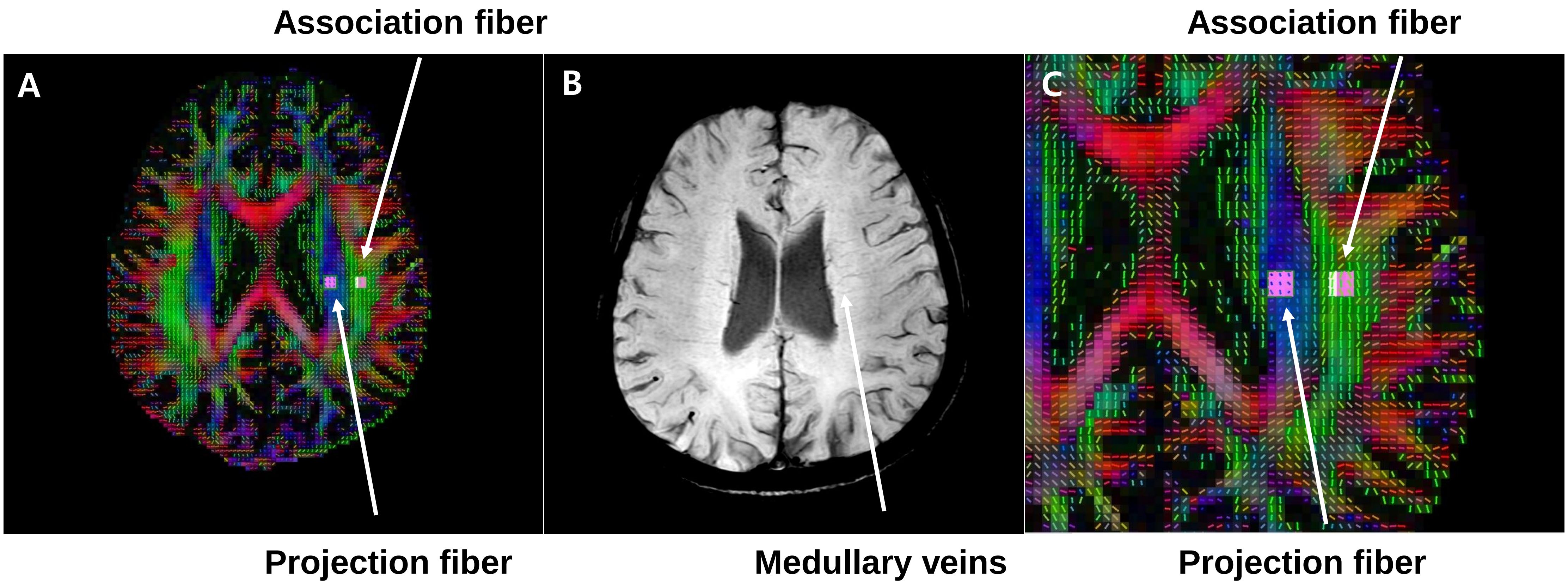

Suppl. 1. Region of interest for obtaining DTI-ALPS index

We identify region in which the lateral projections of the medullary veins traced orthogonal to the primary diffusion directions (A) (B), and the diffusion parameters are calculated in the projection and association tracts after magnifying the area (C).
