# Supplementary material for: Glymphatic Dysfunction in Patients With End-Stage Renal Disease
Source: Front Neurol. 2022 Jan 25;12:809438. doi: 10.3389/fneur.2021.809438 (PMC8821099; doi:10.3389/fneur.2021.809438)
Supplement: Supplementary file 2 [file Data_Sheet_2.PDF]

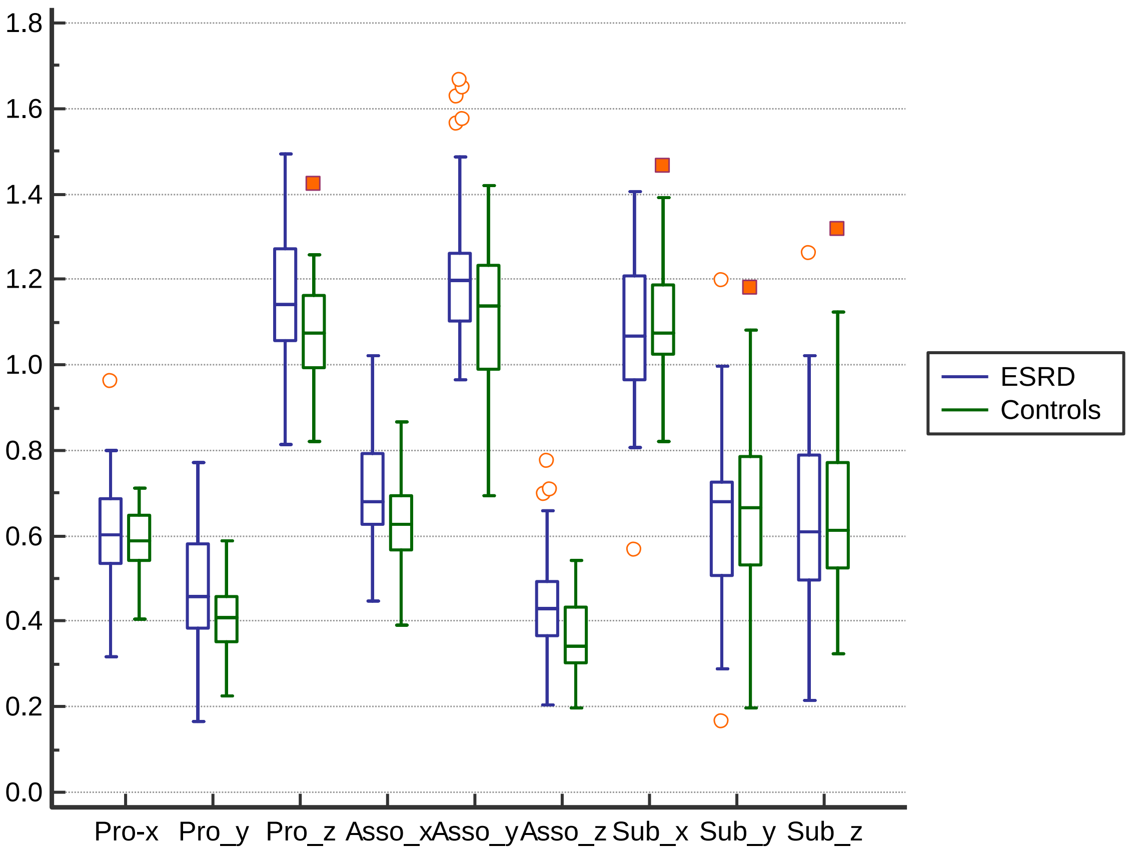

Suppl. 2. Diffusivity along the axis in the fibers

Diffusivities along the x-axis, y-axis, and z-axis in the association fiber and along the y-axis and z-axis in the subcortical fiber in patients with ESRD appear significantly different from those in the healthy controls ($\times 10^{-3}$).

ESRD: end-stage renal disease; Pro_x: diffusivity along the x-axis in the projection fiber; Pro_y: diffusivity along the y-axis in the projection fiber; Pro_z: diffusivity along the z-axis in the projection fiber; Asso_x: diffusivity along the x-axis in the association fiber; Asso_y: diffusivity along the y-axis in the association fiber; Asso_z: diffusivity along the z-axis in the association fiber; Sub_x: diffusivity along the x-axis in the subcortical fiber; Sub_y: diffusivity along the y-axis in the subcortical fiber; Sub_z: diffusivity along the z-axis in the subcortical fiber.
